# Nitric oxide mediates metabolic functions in the bivalve *Arctica islandica* under hypoxia

**DOI:** 10.1371/journal.pone.0232360

**Published:** 2020-05-07

**Authors:** Julia Strahl, Doris Abele

**Affiliations:** 1 Alfred Wegener Institute Helmholtz Centre for Polar and Marine Research, Bremerhaven, Germany; 2 Helmholtz Institute for Functional Marine Biodiversity at the University of Oldenburg, Oldenburg, Germany; 3 Institute of Biology and Environmental Sciences, Carl-von-Ossietzky University Oldenburg, Oldenburg, Germany; Biodiversity Research Center, TAIWAN

## Abstract

The free radical nitric oxide (NO) is a powerful metabolic regulator in vertebrates and invertebrates. At cellular concentrations in the nanomolar range, and simultaneously reduced internal oxygen partial pressures (*p*O_2_), NO completely inhibits cytochrome-*c*-oxidase (CytOx) activity and hence mitochondrial- and whole-tissue respiration. The infaunal clam *Arctica islandica* regulates *p*O_2_ of hemolymph and mantle cavity water to mean values of <5 kPa, even in a completely oxygen-saturated environment of 21 kPa. These low internal *p*O_2_ values support a longer NO lifespan and NO accumulation in the body fluids and can thus trigger a depression of metabolic rate in the clams. Measurable amounts of NO formation were detected in hemocyte cells (~110 pmol NO 100^−1^ hemocytes h^-1^ at 6 kPa), which was not prevented in the presence of the NO synthase inhibitor L-NAME, and in the gill filaments of *A*. *islandica*. Adding a NO donor to intact gills and tissue homogenate significantly inhibited gill respiration and CytOx activity below 10 kPa. Meanwhile, the addition of the NO-oxidation product nitrite did not affect metabolic rates. The high nitrite levels found in the hemolymph of experimental mussels under anoxia do not indicate cellular NO production, but could be an indication of nitrate reduction by facultative anaerobic bacteria associated with tissue and/or hemolymph biofilms. Our results suggest that NO plays an important role in the initiation of metabolic depression during self-induced burrowing and shell closure of *A*. *islandica*. Furthermore, NO appears to reduce mitochondrial oxygen radical formation during surfacing and cellular reoxygenation after prolonged periods of hypoxia and anoxia.

## Introduction

The free radical nitric oxide (NO) is an important intra- and extracellular signalling molecule and its role as a physiological messenger has been functionally conserved between invertebrates and mammals [1]. NO has various biological roles in the nervous, cardiovascular, and immune systems of marine invertebrates, where it is involved in processes such as immune defense, environmental stress response, as well as hemocyte aggregation and regulation of blood pressure [1]. In the blue mussel *Mytilus edulis* and the carpet shell clam *Ruditapes decussates*, NO produced by hemocytes leads to bacterial clumping [2] and to the death of invading pathogens [3]. A recent study highlights the basal function of NO in improving perfusion of hypoxic invertebrate tissues, which could be a key mechanism of tolerance to environmental oxygen (O_2_) shortage and variability (e.g. in the intertidal) [4]. It has been shown that under hypoxic conditions, NO is generated in the muscle cells surrounding the hemolymphatic vessels of gill filaments in *M*. *edulis*, causing an opening of the blood vessel to functionally stabilize whole animal respiration as *p*O_2_ declines [4]. NO has also been recognized as a potent mitochondrial regulator in vertebrate and invertebrate cells, where it reduces the oxygen affinity of cytochrome-*c*-oxidase (CytOx), the terminal electron acceptor of the mitochondrial electron transport chain [4, 5, 6]. NO binding to the enzyme is reversible and competitive with oxygen and, therefore, depends on the cellular oxygen concentration [5, 7]. Furthermore, NO can interact with respiratory complex I (NADH: ubiquinone oxidoreductase) by various mechanisms, leading to non-reversible inhibition of mitochondrial respiration [8]. High enough NO levels cause tiered- to complete inhibition of cellular respiration, depending on the applied NO concentration and tissue partial pressure of O_2_ (*p*O_2_) [4,9]. Hence, NO plays a key role in the process of metabolic rate depression (MRD) in both vertebrate and invertebrate species that are entering a dormant or quiescent state (e.g., hibernation, torpor), or experiencing environmental hypoxic or anoxic conditions (e.g., in intertidal and subtidal habitats).

NO can be generated enzymatically by cytosolic (and presently unconfirmed in marine invertebrates: mitochondrial) nitric oxide synthase (NOS) [10] or by xanthine-oxidoreductase [11, 12] in animal cells. NOS-like activity has been reported for marine, fresh water and terrestrial molluscs [13], i.e. in the digestive gland of *Mya arenaria* [14], in hemocytes of the freshwater snail *Viviparus ater* [15], and in the central nervous system of the snail *Pleurobranchea californica* [16]. NO can be further produced by acidic reduction of nitrite in mammal- and ectotherm tissues (reductive sequence: NO_2_^-^ + H^+^ ↔ HNO_2_ / 2 HNO_2_ ↔ H_2_O + N_2_O_3_ / N_2_O_3_ ↔ NO + NO_2_) [16, 17, 18].

The infaunal clam *Arctica islandica* is capable of self-induced burrowing and MRD [19, 20, 21]. Hence, periods during which the clams are respiring at the surface are interspersed with periods when the animals are burrowed several centimetres deep in the sediment, with shells closed, whereupon the internal *p*O_2_ drops to 0 kPa [19,21]. Marine molluscs down-regulate hemolymph- and mantle cavity water *p*O_2_ even in fully oxygenated environments (21 kPa), which supports a longer half-life of NO at the physiologically low *p*O_2_ of 1–5 kPa in their body fluids [22]. The more dissolved oxygen in an aqueous medium, the more rapidly NO is oxidized to nitrite according to the reaction: NO + O_2_ + 2 H_2_O → 4 NO_2_^-^ + 4 H^+^ [23].

Moroz and colleagues [10] reported a variety of NO concentrations measured in molluscan central nervous systems, between 10 and 300 nM, and attributed these to the rather low *p*O_2_ in the molluscan hemolymph of 20–40 torr (2.5–5 kPa) in non-specified marine molluscs and 5–12 torr (0.7–1.6 kPa) in fresh water snails [10]. In *A*. *islandica* from the German Bight and Kiel Bight, the mean mantle cavity water *p*O_2_ is < 5 kPa in fully oxygenated water [20, 24]. Thus, NO may play a role in the self-induced MRD in *A*. *islandica*, as prolonged burrowing or shell closure lead to a decline in internal *p*O_2_, promoting the stabilization of NO in body fluids and tissues and, consequently, a reduction of mitochondrial respiration by reversible CytOx inhibition.

We hypothesized that NO plays a central role in the *p*O_2_-dependent inhibition of mitochondrial respiration in *A*. *islandica*. Our first aim was therefore to examine the formation of NO in hemocytes and gill tissue of the ocean quahog, *A*. *islandica*, under declining experimental *p*O_2_ conditions between 16 (normoxia) and 2 kPa (hypoxia). To establish whether NO formation in *A*. *islandica* is catalyzed enzymatically, NO production was measured in the presence and absence of the NOS inhibitor (L-NAME). In a second set of experiments, we examined the effect of chemically produced NO on gill respiration and CytOx activity, during phased reduction of *p*O_2_ (16 kPa-2 kPa) to characterize the inhibitory effect of NO on mitochondrial and whole tissue oxygen uptake. To test whether NO-oxidation products can indicate NO production, nitrite and nitrate contents were determined in the hemolymph of bivalves after exposure to normoxic, hypoxic, and anoxic conditions.

## Materials and methods

### Bivalve collection and maintenance

**Fig 1:**
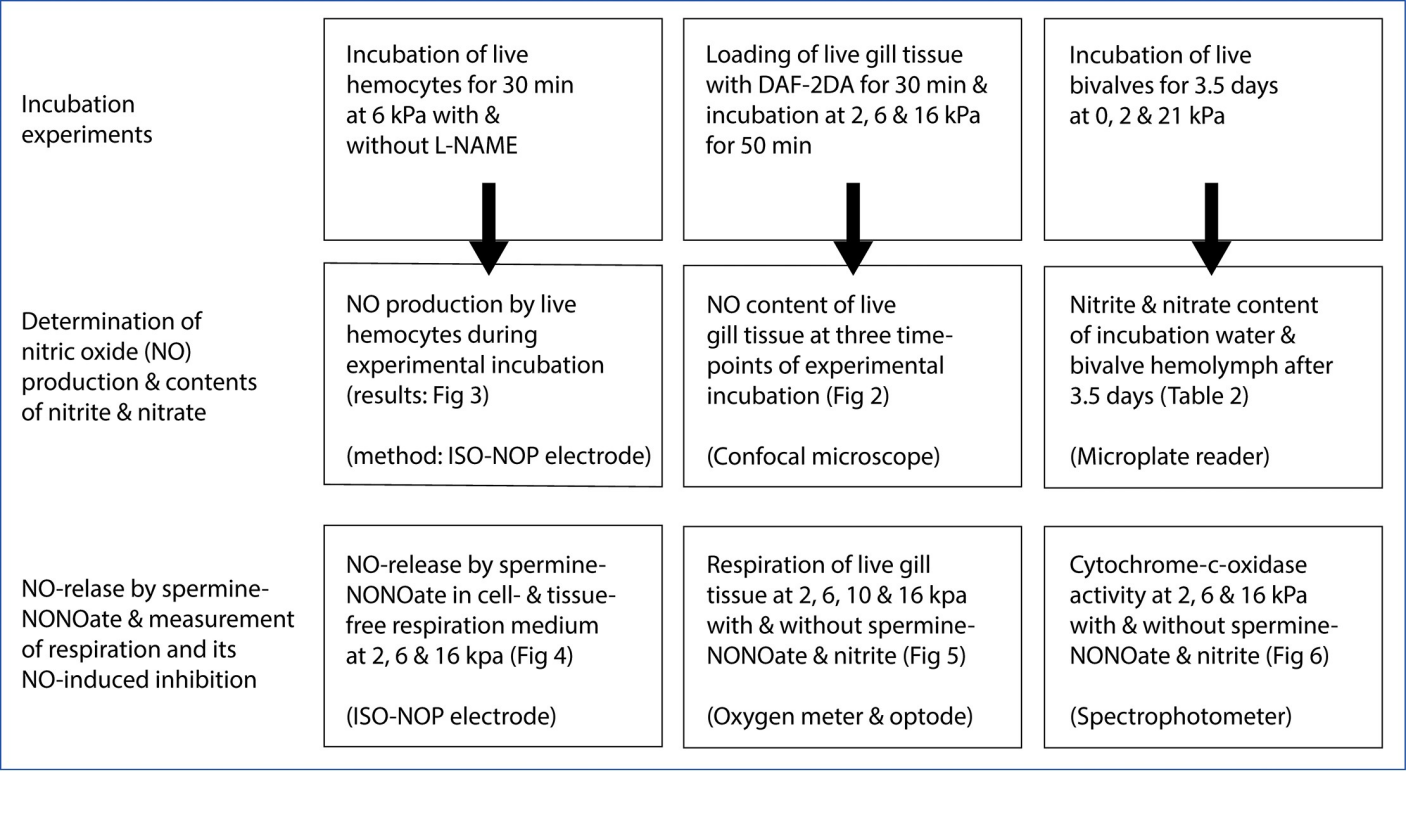
Overview of incubation experiments and laboratory analyses.

Because of the mechanistic, rather than quantifying, approach of the present study, medium-sized *A*. *islandica* (shell height: 40–90 mm) from different source populations were used to conduct experiments and analyses (Fig 1). No permits were required for the collection as this bivalve species is not threatened by extinction and stocks are used commercially. In May, 2008, *A*. *islandica* were collected with a trawl net around Helgoland in the German Bight (54°09.05’N, 07°52.06’E, water depth: 40 − 45 m, surface water temperature: 12°C), and in August 2008 northeast of Iceland (66°01.44’N, 14°50.91’W, water depth: 8−15 m, surface water temperature: 9°C). Bivalves were then transported in cooled containers to the Alfred Wegener Institute Helmholtz Centre for Polar and Marine Research in Bremerhaven, where they were maintained at 10°C and 33 PSU in a 60L tank containing re-circulating seawater with 10 cm of pea-gravel sediment (2–3 mm grain size). For four weeks prior to the start of the experiments, the animals were allowed to acclimatize to aquarium conditions. They were fed once a week with a mixture of live phytoplankton (DT´s plankton farm, USA, *Nannochloropsis occulata*, *Phaeodactylum tricornutum* and *Chlorella sp*., 3 ml bivalve^-1^ wk^-1^) and at least 48 h were allowed between feeding and the start of the analysis to avoid possible interference of any nutrient-induced increase in metabolic rates. Water quality was assessed weekly using Nanocolor Tube Tests (Machery-Nagel GmbH & Co. KG, Germany), and the water was changed when values of ammonium and nitrate exceeded 0.4 mg l^-1^and 0.2 mg l^-1^, respectively.

### Nitric oxide production by hemocytes

Five *A*. *islandica* were used as hemolymph donators to measure hemocyte NO production. Shell closure was prevented by inserting a metal bar (3 mm thick and 3 cm long) between the two bivalve shells. Between 6 and 10 ml of hemolymph were slowly drawn from the adductor muscle of each specimen with a sterile needle and a 10 mL-syringe, and centrifuged for 10 min at 450 g and 10°C. The supernatant was discarded and the pellet containing the hemocytes was re-diluted in 2.5 ml of 60 mM Tris-HCl buffer-100 mM KCl (pH 7.0) to concentrate the hemocytes. A 0.1 ml subsample of each sample solution was used to count hemocytes in a hemocytometer (Neubauer improved counting chamber) and thus determine cellular density in the hemolymph. Subsequently, two 1.2 ml samples of the hemocytes were transferred to two temperature- and *p*O_2_-controlled glass chambers, adjusted to 10°C and a 6 kPa *p*O_2_ using a water bath (Julabo FP 40, Germany) and a gas mixing pump (Wösthoff GmbH, Bochum, Germany). To test for “NOS-like” NO generation, the concentrated hemocyte samples in both glass chambers were supplemented with 0.1 mM NADPH, 5 mM MgCl_2_, 1 mM CaCl_2_, 1 mM L-arginine and 0.25% antibiotic antimycotic (GIBCO^™^ Antibiotic-Antimycotic (100X), Invitrogen). Additionally, 5 mM of the NOS inhibitor L-NAME were added to the incubation mixture in chamber two, but not in chamber one. As a large hemolymph volume was required to harvest enough cells for the measurement, each bivalve could be sampled only once for this analysis. Therefore, no replicate measurements with and without L-NAME were conducted for individual animals. Each chamber was equipped at the bottom with a magnetic stirrer turning gently below a net fixed by a plastic ring to protect the hemocytes, and each chamber was closed with a stopper. The NO produced by hemocytes of individual bivalves was measured for 30 min with and without L-NAME, using a free radical analyzer (Apollo 4000, World Precision Instruments, USA) equipped with an amperometric NO-electrode (ISO-NOP, World Precision Instruments, USA), introduced into the chambers through a hole in the stopper. Prior to each measurement, the electrodes were calibrated at 10°C based on chemical generation of NO following the manufacturer´s instructions. The NO production rates were given in pmol NO h^-1^ 100^-1^hemocytes. Finally, NO production rates in hemolymph of *A*. *islandica in vivo* were calculated based on known hemocyte densities of ~140.000 hemocyte cells ml^-1^ hemolymph [25].

### Nitric oxide production by gill tissue

**Fig 2:**
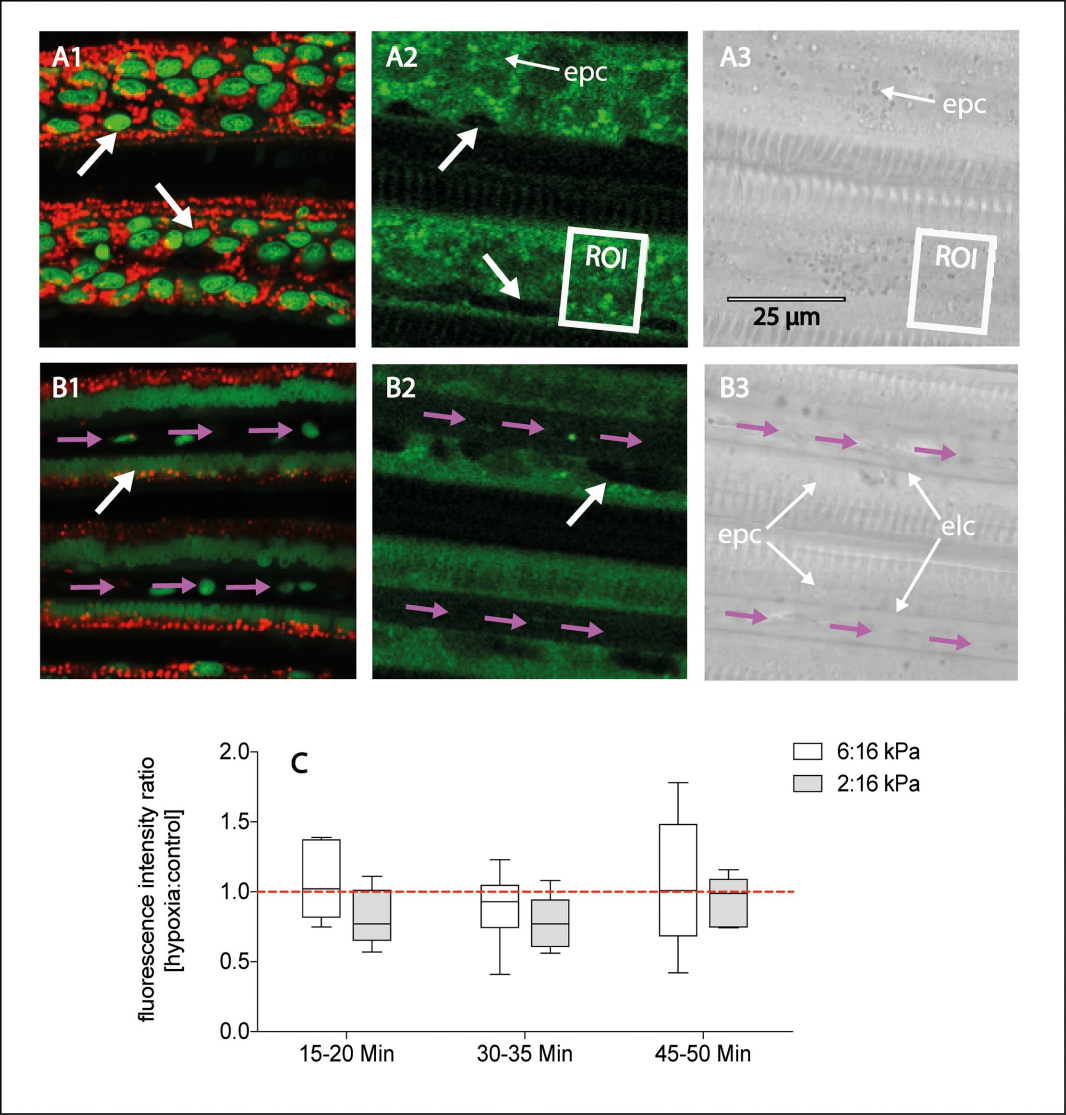
Imaging of gill filaments of *Arctica islandica* 5 μm above the z-depth **(A)**, and in the z-depth of the blood vessel **(B)**; Nitric oxide (NO) formation in gills is expressed as the DAF-2T fluorescence intensity ratio between hypoxic and control *pO*_*2*_ (C; n = 5–6). A1&B1: Overview of gill-filament structure (fluorescence image), showing nuclei in green (Syto-13 staining) and mitochondria in red (Mitrotracker Deep Red 633 staining); A2&B2: Representative NO formation in gill filaments indicated as green fluorescence (DAF-2T); A3&B3: Transmission image. The white box represents the region of interest (ROI) in the gill filaments, one layer above the z-depth analyzed for NO formation; bold white arrows indicate nuclei; pink arrows indicate blood vessel in the z-depth of the filament; epc = epithelial cells; elc = endothelial cells.

For each bivalve analyzed, six freshly excised gill pieces were transferred to 5 ml incubation medium (0.2 μm filtered seawater, 15 mM Na-HEPES, 0.5 mM glucose), supplemented with DAF-2DA (Sigma D224, 5 mM in DMSO, Table 1) and adjusted to 10°C and 16 kPa *p*O^2^ using a water bath (Julabo FP 40, Germany) and a gas mixing pump (Wösthoff GmbH, Bochum, Germany). After 30 min of loading time, three gill pieces each were transferred to 5ml of fresh incubation medium adjusted to 10°C and control (16 kPa) and hypoxic conditions (2 kPa or 6 kPa), respectively. After 15–20 min, 30–35 min and 45–50 min of exposure to different *p*O_2_, imaging of NO-induced DAF-2T fluorescence in live gill filaments was conducted using a Leica TCS SPII confocal microscope (Leica Microsystems CMS GmbH, Wetzlar, Germany) equipped with a multiphoton laser (MaiTai-DeepSee, Spectra-Physics, Newport Corp., Table 1) and a 40x optical objective. Each gill filament was placed onto a microscope slide with 200 μl incubation medium of the respective *p*O_2_ and imaged within a few seconds. In order to locate the nuclei and the mitochondria in the gill filaments (Fig 2A1 and Table 1) and localize the tissue structures centrally involved in NO-formation, additional gill pieces were stained with SYTO-13 (Molecular Probes S7575, 5 mM in DMSO) and Mitotracker Deep Red 633 (Molecular Probes M-22426, 1 mM in DMSO). To avoid photo-bleaching, an initial short period (> 5 sec) of low-resolution scanning (512 x 512 pixels) was applied for focal adjustment. Then, for image analysis and NO fluorescence quantification, a minimum of five scans with higher resolution (1024 x 1024 pixels) were conducted across the surface of each gill piece. For each of the scans taken, a total of three square cross sections (= regions of interest, ROI) were plotted across the gill filaments (Fig 2A2) in the layer with the highest fluorescence intensity, which was 5 μm above the z-depth containing the blood vessel (Fig 2B). The fluorescence intensity of each ROI was quantified using the Leica LAS AF-TCS-SPS Lite software (Leica Microsystems CMS GmbH, 2011, Version 2.6.0) and the average NO formation over time was calculated as the average DAF-2T fluorescence intensity ratio between hypoxic and control *pO*_*2*_ (6:16 kPa and 2:16 kPa) after 15–20 min, 30–35 min, 45–50 min.

**Table 1:** Analysis conditions for the individual dyes.

Dye	Mechanism of function	Final Concentration (μM)	Incubation time (min)	Excitation (nm)*	Emission (nm)
DAF-2DA	DAF-2 is formed by intracellular hydrolization of its ester bonds by esterases. It reacts with nitrosonium cation, forming the fluorescent DAF-2T.	20	30	488	505–525
MitoTracker Deep Red 633	Molecule becomes fluorescent once accumulated in the lipid environment of the mitochondria.	1	60	633	640–680
Syto-13	Molecule exhibits a green fluorescence upon binding to nucleic acid.	10	60	488	500–520

*intensity of argon laser during measurements: 30%

### Incubation experiments and measurement of nitrite and nitrate content

*Arctica islandica* were incubated individually in 3-L flasks filled with natural seawater at constant water temperature and salinity (10°C, 33 PSU) and three different *p*O_2_ as described in Strahl et al. [26]. Before the experiment, animals were kept without food for three days to reduce eutrophication of the incubation water by feces and microbial contamination. Specimens were incubated in normoxic (21 kPa), hypoxic (2 kPa), or anoxic (0 kPa) sea water, which was *p*O_2_-adjusted using gas mixtures of nitrogen and oxygen (Air Liquide, Germany). Over the time course of the experiment, the siphon status of each bivalve was checked visually three times a day. After 3.5 days of incubations, shell closure of each bivalve was prevented by inserting a metal bar (3 mm thick and 3 cm long) between the two shells. Hemolymph was gently drawn from the adductor muscle of each individual with a sterile needle and a 10 ml-syringe, and a 10 ml sample of the incubation water was taken from each flask. Nitrite (NO_2_^-^) and nitrate (NO_3_^-^) content in aquarium water and hemolymph were measured with the Griess method, following Misko et al. [27] and Verdon et al. [28]. Each sample was pre-incubated with and without nitrate reductase, to be able to determine nitrite only, as well as the sum of nitrite + nitrate (∑NiNa). To convert nitrate to nitrite, 50 μl of samples or standards (125 μM nitrite or nitrate) was mixed with 10 μl NADPH and 40 μl of freshly prepared reaction mixture (final concentrations: 1 μM NADPH, 500 μM glucose-6-phosphate, 160 U/L glucose-6-phosphat dehydrogenase, 80 U/L nitrate reductase and 14 mM sodium phosphate buffer, pH 7.2) and incubated for 45 min at 20°C. The 100 μl reaction solutions were mixed with 100 μl each of 1% (v/w) sulfanilamide in 5% phosphoric acid (SA) and 0.1% (v/w) N-(1-naphthyl)ethylenediamine HCL (NED), respectively. Samples, blank, and standards were incubated for 10 min at 20°C to determine the nitrite content at 540 nm in a microplate reader (Sunrise, Tecan, Germany). Nitrite concentrations of the samples were calculated using the standard curve after subtracting the respective blank value.

### Measurement of gill respiration and its NO-induced inhibition at different *p*O_2_

Before the experiment, bivalves were kept without food for three days, to eliminate the effect of specific dynamic action (SDA) on gill respiration [29]. Gills of *A*. *islandica* were freshly dissected and transferred to cooled respiration buffer (450 mM NaCl, 10 mM KCl, 20 mM MgCl_2_, 10 mM HEPES, 1 mM EGTA, 0.5 mM DTT, 0.055 mM glucose, pH 7.4) following Strahl et al. [20]. Gills of each test animal were cut into two pieces of 15–25 mg wet mass (WM) and incubated for 2 h in respiration buffer at 10°C and a *pO*_*2*_ of 2 kPa, 6 kPa, 10 kPa or 16 kPa, adjusted with a gas mixing pump (Wösthoff GmbH, Bochum, Germany). After incubation, one piece of gill from each individual was transferred to a temperature-controlled respiration chamber filled with 1.1 ml respiration buffer at 10°C and the respective test *p*O_2_. As described in Strahl et al. [26], oxygen consumption rate of each gill piece was measured for 30 min at 2 kPa, 6 kPa, 10 kPa or 16 kPa using single-channel Microx TX-3 oxygen meters equipped with oxygen needle-optodes (PSt1-L5-TF, Precision Sensing GmbH, Germany) that had been calibrated to 21 and 0 kPa. The second gill piece of each specimen was measured in parallel in another chamber at 10°C. In this chamber, gill respiration was inhibited with spermineNONOate at the respective test *p*O_2_. After *p*O_2_ equilibration in the chamber, spermineNONOate was injected to release NO. The concentration of spermineNONOate needed for complete (= 100%) inhibition of gill respiration for a 20 min time span was measured at each experimental *p*O_2_. WM of each gill piece was measured and respiration rates were calculated as nmol O_2_ min^-1^ ml^-1^ mg^-1^ WM. The inhibitory effect of nitrite on gill respiration was tested by adding 575 μM nitrite (which is well above values of ~ 40 μM measured in hemolymph of *A*. *islandica* in this study) to pieces of excised gill that were incubated at either 6 kPa or 10 kPa.

In a separate experiment using the ISO-NOP-electrode, we examined the NO yield for each spermineNONOate concentration required to achieve complete inhibition of gill respiration in the above experiments. Furthermore, to calculate the rate constant (K) and the steady state of NO ([NO]_steady state_) in the respiration buffer, we added 197 μM spermineNONOate to 1.1 ml respiration buffer at 10°C and measured the NO concentration after 5 min, 10 min, 15 min and 20 min with the ISO-NOP-electrode at 2 kPa, 6 kPa, and 16 kPa, respectively. The parameters (K and [NO]_steady state_) were calculated in Python by fitting the *p*O_2_-dependent NO concentrations with the diffusion equation in the form of: [NO] = [NO]_steady state_ * (1 -e^-Kt / [NO]steady state^).

### Measurement of in vitro CytOx activity and its NO-induced inhibition at different *p*O_2_

Frozen gill tissue was ground in liquid nitrogen and homogenized with a glass homogenizer (Nalgene) in Tris-HCl buffer (20 mM Tris-HCl, 1 mM EDTA, 0.01% (v/v) Tween® 20, pH 7.4) 1:3 (w/v). Homogenates were centrifuged for 10 min at 1000 ×*g* and 2°C and incubated for 30 min at 2 kPa, 6 kPa or 16 kPa, respectively, adjusted by a gas mixing pump. CytOx activity was determined after Moyes et al. [30] by measuring the oxidation rate of fully reduced cytochrome *c* at 550 nm in 20 mM Tris HCl buffer with 0.05% Tween 20, pH 8.0. Activity was calculated using the extinction coefficient ε550mM (= 19.1 mM^–1^ cm^–1^) after Hardewig et al. [31]. In a second cuvette the spermineNONOate was added to the homogenate to achieve 50% CytOx activity inhibition at the two hypoxic *p*O_2_ (2 kPa, 6 kPa). The inhibitory effect of nitrite was tested by adding 575 μM nitrite to the homogenates.

### Statistical analysis

Statistical analyses were performed with GraphPad Prism Software (Version 6.07, USA). All data sets were tested for normality (Kolmogorov–Smirnov test) and homogeneity of variances (Bartlett’s test) before testing for differences in NO production with or without the NOS-inhibitor L-NAME, for *p*O_2_ specific- and metabolic-state specific differences in nitrite and nitrate content, or for differences in gill respiration and CytOx activity with and without chemically generated NO at and between each specific *p*O_2_. Results not meeting statistical significance (P > 0.05) are not shown.

## Results

### NO production rates of hemocytes

**Fig 3:**
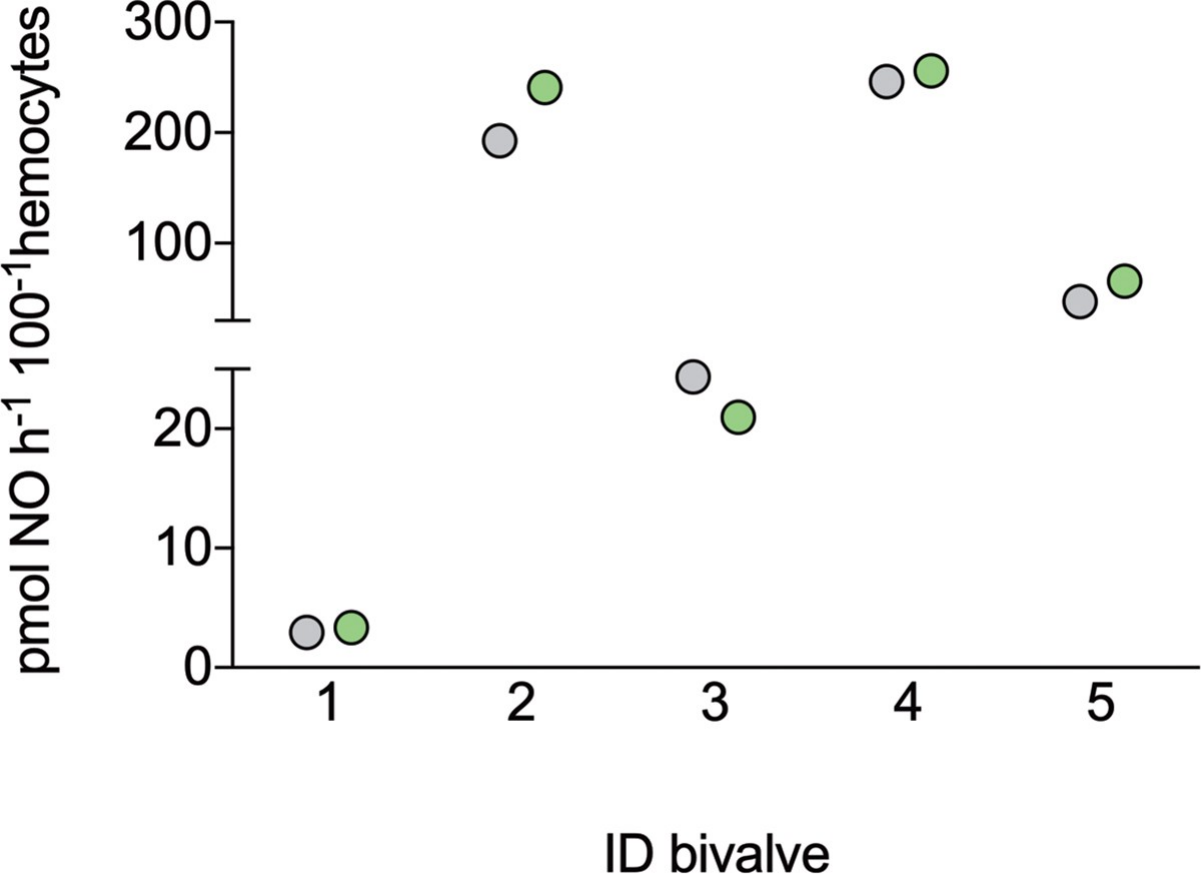
Nitric-oxide formation rates of live hemocyte cells of five individual *Arctica islandica* (ID 1–5) at 6 kPa *p*O_2_ with (green circles) and without the nitric oxide synthase inhibitor L-NAME (grey circles). Measurements conducted with an ISO-NOP electrode.

NO production by *A*. *islandica* hemocyte cells at 6 kPa was highly variable between individual bivalves, with a mean of 110 pmol NO h^-1^ 100^−1^ hemocytes (Fig 3). No significant differences were detected between hemocytes incubated with or without the NOS-inhibitor L-NAME, indicating NOS-independent NO formation, reproducible between two measurements. Calculated NO formation rates in hemolymph of *A*. *islandica in vivo* (based on known hemocyte densities) [25] were between 42 and 358 μmol NO h^-1^ ml^-1^, with a mean of 154 μmol NO h^-1^ ml^-1^.

### NO formation in gill tissue

Confocal images of gill filaments were analyzed for NO (= DAF-2T fluorescence intensity) at 5 μm above z-depth (= above the blood vessel), as this was the layer of strongest fluorescence intensity (Fig 2A2). At all *p*O_2_ examined, DAF-2T fluorescence was most pronounced in mitochondria and epithelial cells of gill filaments. In contrast, the nuclei (Fig 2A1) appeared black in the DAF-2T fluorescence images (Fig 2A2). Throughout the experimental timeline and for both hypoxic treatments, fluorescence intensities were similar in gill pieces exposed to control (16 kPa) or hypoxic conditions (6 kPa, 2 kPa), leading to fluorescence intensity ratios (hypoxia:normoxia) close to 1 (Fig 2C).

Images taken directly at the z-depth containing the blood vessel showed the absence of DAF-2T staining in the endothelial cells surrounding the blood vessel (Fig 2B2).

### Nitrite and nitrate contents of hemolymph and incubation water

All bivalves survived experimental exposure to different *p*O_2_ for 3.5 days. We observed their siphons to be permanently open during hypoxic (2 kPa) and anoxic (0 kPa) incubations, whereas bivalves exposed to normoxia (21 kPa) alternately opened and closed siphons.

After 3.5 days, *p*O_2_-dependent differences were observed for ∑NiNa, nitrite, and nitrate concentrations in incubation water and bivalve hemolymph (Table 2). In the incubation water, ∑NiNa decreased in a *p*O_2_-dependent manner, while in the hemolymph, values were 30–40% lower under hypoxic, compared to normoxic and anoxic conditions. Interestingly, nitrate decreased significantly (>6-fold) at 0 kPa *p*O_2_ in incubation water and hemolymph, while hemolymph nitrite increased correspondingly (8-fold), indicating denitrification processes occurring in anoxic bivalves (Table 2).

**Table 2:** Concentration of nitrite, nitrate and the sum of both (∑NiNa) in incubation water (A.) and hemolymph of *Arctica islandica* (B.) after 3.5 days of normoxic (21 kPa), hypoxic (2 kPa), or anoxic (0 kPa) incubation. Mean ± SD, *n* = 6–10.

	21 kPa	2 kPa	0 kPa
*A*. *Incubation water*			
∑NiNa (μM)	34.21 ± 6.68	**22.02 ± 3.65** *	**8.44 ± 3.93** *
Nitrite (μM)	1.75 ± 1.07	0.83 ± 0.80	3.63 ± 4.33
Nitrate (μM)	32.46 ± 7.25	**21.19 ± 3.91** *	**5.29 ± 4.61** **
*B*. *Bivalve hemolymph*			
∑NiNa (μM)	37.90 ± 7.37	**26.41 ± 2.44** ^**#**^	38.01 ± 7.08
Nitrite (μM)	4.48 ± 2.51	4.60 ± 2.08	**37.08 ± 9.45** *
Nitrate (μM)	33.42 ± 6.84	21.82 ± 2.32	**2.30 ± 2.74** *

Significant effects in bold

* and

** ∑NiNa and nitrate in incubation water differ significantly between normoxic, hypoxic, and anoxic conditions (One-way ANOVA P < 0.001 and P < 0.0001, Tukey P < 0.01 and P < 0.001).

^#^ ∑NiNa in hemolymph differs significantly between hypoxic *vs*. normoxic and anoxic conditions (One-way ANOVA P < 0.0001, Tukey P < 0.01).

^##^ Nitrate and nitrite in hemolymph differ significantly between anoxic vs. normoxic and hypoxic conditions, and between anoxic hemolymph *vs*. anoxic aquarium water (One-way ANOVA P < 0.0001, Tukey P < 0.001).

### NO release by spermineNONOate

**Fig 4:**
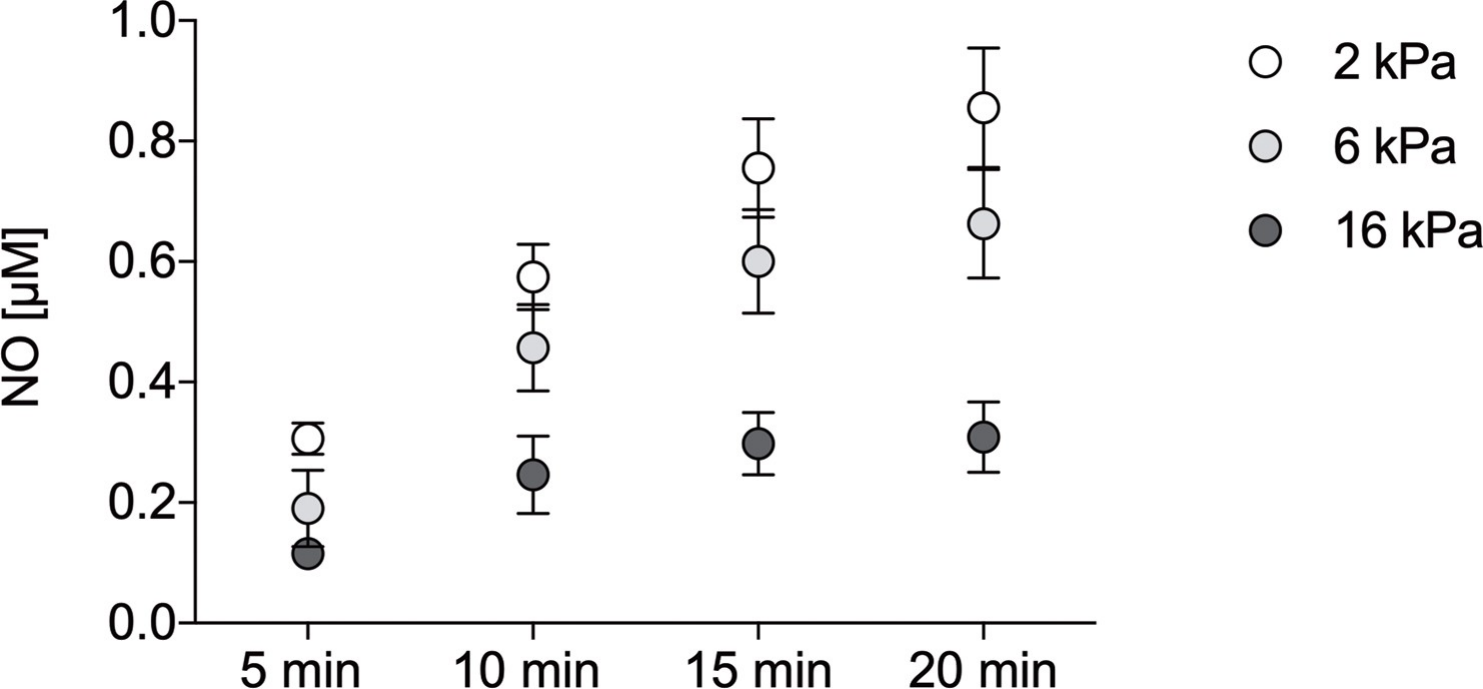
Average Nitric Oxide (NO) concentrations over time in cell- and tissue-free respiration medium containing 197 μM spermineNONOate at three different *pO*_*2*_. N = 3 per *pO*_*2*_; measurements conducted with an ISO-NOP electrode.

Prior to the gill-respiration study, the *p*O_2_-dependent NO concentration released by the NO-donor spermineNONOate was measured. The addition of 197 μM of spermineNONOate to the cell- and tissue-free respiration medium yielded higher NO concentrations at lower *p*O_2_. The average NO concentrations after 20 min (Fig 4) and calculated steady states of NO were 2-3-fold lower at 16 kPa compared to hypoxic *p*O_2_ (2 and 6 kPa; Table 3).

**Table 3:** Calculated rate constant (K) and steady state of NO ([NO]_steady state_) in cell- and tissue-free respiration medium containing 197 μM spermineNONOate at three different *pO*_*2*_.

	16 kPa	6 kPa	2 kPa
	(240 μM O_2_)	(90 μM O_2_)	(30 μM O_2_)
[NO]_steady state_ in μM NO	0.372	1.071	1.212
K in μM NO min^-1^	0.036	0.055	0.076
Error (in μM NO)	0.032	0.068	0.030

### Gill-respiration rates and inhibition by NO

**Fig 5:**
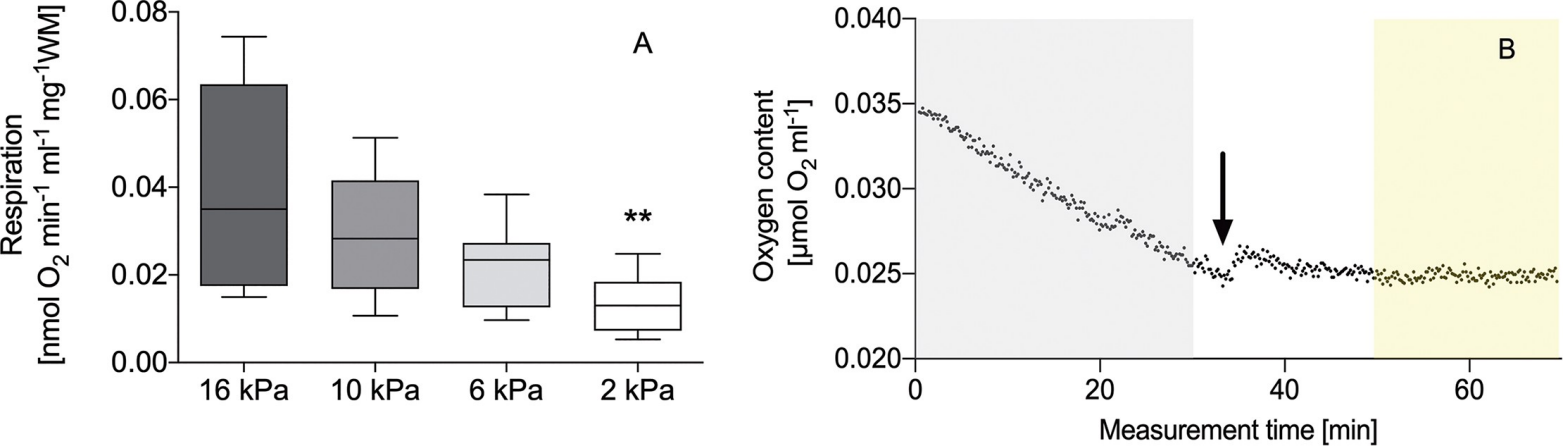
**A.** Gill-respiration rates of *Arctica islandica* at four different *p*O_2_ without spermineNONONate; n = 9–12. ** Respiration rates differ significantly between 16 kPa and 2 kPa (One-way ANOVA and Tukey P < 0.005). **B:** Example of oxygen consumption by live gill tissue at 2 kPa under control conditions (without spermineNONONate, grey background), and under complete NO-induced inhibition of gill respiration (0.00 nmol O_2_ min^-1^ ml^-1^ mg^-1^ WM) within 20 min (yellow background) after adding spermineNONONate to the measurement (black arrow). Measurements conducted with an ISO-NOP electrode.

Gill respiration of *A*. *islandica* showed an oxyconform pattern, with significantly declining respiration rates (-65%) between 16 kPa and 2 kPa (Fig 5A). Respiration was completely inhibited at 2, 6 and 10 kPa (= 0.00 nmol O_2_ min^-1^ ml^-1^ mg^-1^ WM; n = 3–4) by NO in in the low μM range (Table 4) after adding spermineNONOate to the medium (example given in Fig 5B). Due to the higher NO oxidation and lower [NO]_steady state_ in the respiration medium at higher *p*O_2_ (Table 3), the concentrations of spermineNONOate required for complete respiration inhibition were three to eight times higher at 6 and10 kPa than at 2 kPa (Table 4). A complete inhibition of gill respiration could not be achieved at 16 kPa with spermineNONOate concentrations of > 5.5 mM.

**Table 4:** Required concentrations of spermineNONOate (SpNONOate), and resulting concentrations of Nitric Oxide (NO), to reach complete inhibition of gill respiration within 20 min at three different *p*O_2_. Mean ± SD; n = 3–4; measurements conducted with an ISO-NOP electrode.

	16 kPa	10 kPa	6 kPa	2 kPa
SpNONOate (mM)	> 5.50	1.18 ± 0.55	0.47 ± 0.37	0.14 ± 0.06
NO (μM)	1.01 ± 0.08	1.90 ± 0.02	1.57 ± 0.21	0.70 ± 0.13

In separate tests, high concentrations of nitrite (575 μM) had no inhibitory effects on gill-respiration rates at any of the applied *p*O_2_ (data accessible at https://doi.org/10.6084/m9.figshare.12071427.v1).

### CytOx activity and inhibition by NO

**Fig 6:**
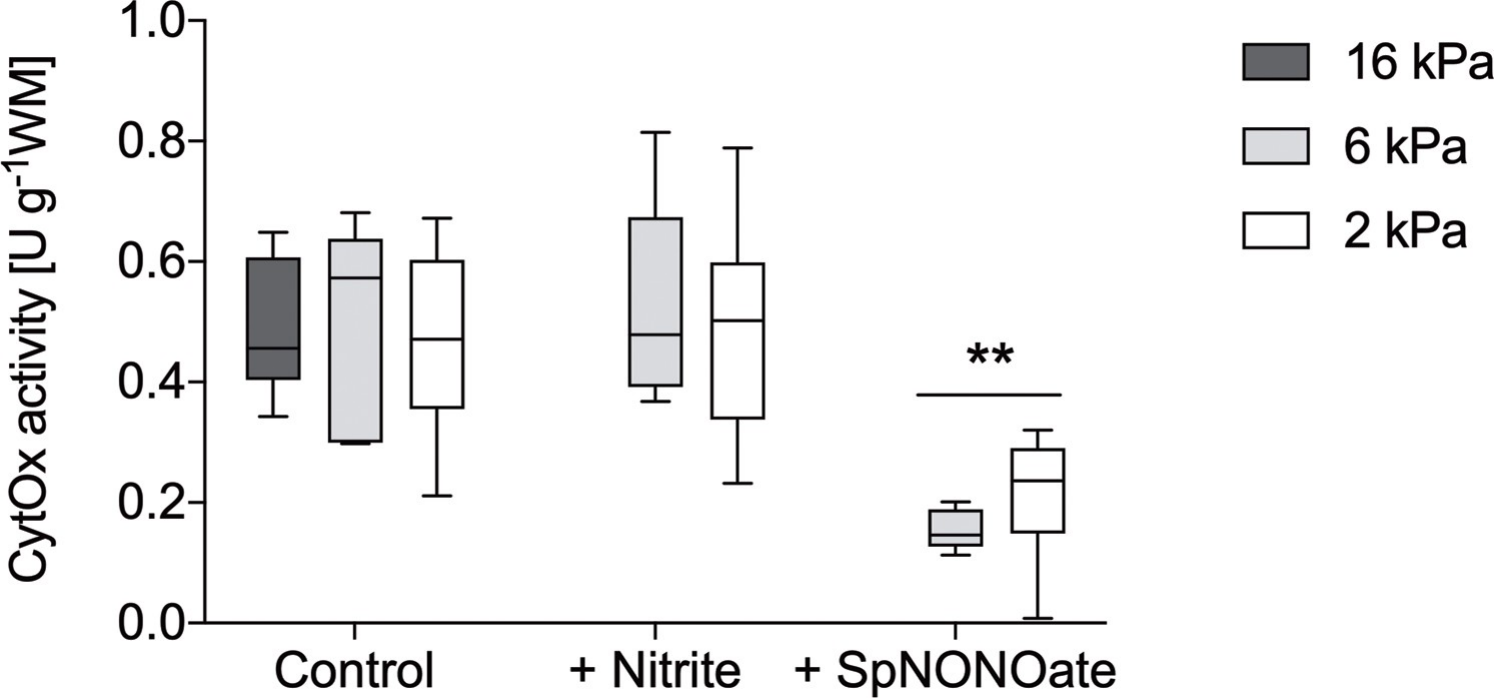
Activity of cytochrome-*c*-oxidase (CytOx) in gill tissues of *Arctica islandica* at three different *p*O_2_ under control conditions (= without spermineNONONate), and in the presence of nitrite or spermineNONOate (SpNONOate). Mean ± SD, *n* = 6–10. ** CytOx activity differs significantly between control and spermineNONOate treatment (One-way ANOVA P < 0.0001, Tukey P < 0.05).

In contrast to gill respiration, CytOx activity at control conditions remained similar between 2 kPa and 16 kPa. After adding high amounts of spermineNONOate (2.7 mM ± 0.8) to the medium, CytOx activity at 2 kPa and 6 kPa was significantly inhibited, by 55% and 70%, respectively (Fig 6). Similar to gill-respiration measurements, 575 μM nitrite had no effect on enzyme activity (Fig 6).

## Discussion

### NO production in live hemocyte cells and gill filaments

Hemocyte cells from five *A*. *islandica* individuals produced variable amounts of NO *in-vitro* under hypoxic conditions. The addition of the nitric oxide synthase (NOS) inhibitor L-NAME did not change the amperometric NO signal, which indicates that NOS is at least not a primary source of NO in the hemocytes. NOS involvement in hemocyte NO production has been tested for different bivalves, with no effect of L-NAME in our study and in *Ruditapes decussatus* [3], another deep burrowing clam species. Potentially, NOS driven NO production is more typical in hemocytes of snails [15] and epifaunal bivalves than for infaunal clams, highly capable of anaerobic survival. In epifaunal species such as the Pacific oyster, 80–85% of hemocyte NO production originates from NADPH-oxidases, including NOS activity [32] and Dailianis [33] found an inhibitory effect of L-NAME on NO production in *Mytilus galloprovincialis* hemocytes (detected as nitrite content). However, nitric oxide production by *A*. *islandica* hemocytes appears to be linked to other enzymatic/non-enzymatic pathways or microbial processes instead of L-NAME-inhibitable NOS, a topic that warrants further investigation. The inter-individual variability in hemocyte NO formation rate in our measurements is difficult to explain, but probably an important component to consider. The differences could be due to the state of oxygenation or the immunological or feeding condition of individual animals. For the experiments we randomly selected animals close to the sediment surface with the shells slightly open. These individuals may have been in various states of hypoxia-reoxygenation after prolonged periods of burrowing, during which shell water and hemolymph become anaerobic and food intake is interrupted for longer periods of time, which may have influenced NO formation rates.

Gill filaments were strongly DAF fluorescent in the confocal images, indicating that NO formation occurs inside the gill cells of *A*. *islandica*. Based on the DAF-2T fluorescence signal, NO formation in gills was clearly confined to epithelial cells and their mitochondria. Interestingly, under normoxic and hypoxic conditions, DAF fluorescence was absent in the muscular endothelium directly around the blood vessel. This is in stark contrast to distinct DAF-2T staining in the muscle cells surrounding the blood vessel in gill filaments of the blue mussel *M*. *edulis* under control conditions [34], which intensifies in hypoxia [4]. The accumulation of NO in *M*. *edulis* gill endothelium was accompanied by stepwise dilation of blood-vessel diameter, which facilitates hemolymph flow and gas exchange at low *p*O_2_ [4]. The conspicuous difference between these bivalve species aligns with their ecological adaptation and lifestyle. The blue mussel is strictly epifaunal and mostly colonizes intertidal and shallow subtidal areas, whereas *A*. *islandica* is a sublittoral sediment dweller. Both have to cope with frequent hypoxia and anoxia, but face different dynamics. In the intertidal, *Mytilus* is exposed to hypoxia and anoxia during low tide [29, 35], which requires fast metabolic adjustment upon shell closure. Here, *p*O_2_-dependent NO generation is a key mechanism to withstand rapid environmental O_2_ fluctuations [4]. Meanwhile, *A*. *islandica* is a hypoxia-adapted species that actively regulates hemolymph and shell water *p*O_2_ at low levels (< 5kPa) through intermittent ventilation [20, 24]. Obviously, there is no need for *Arctica* to conduct rapid adjustments of tissue oxygenation by NO-induced blood-vessel widening.

### *P*O_2_-dependency of NO lifetime: A potential mechanism in metabolic subsidence

**Fig 7:**
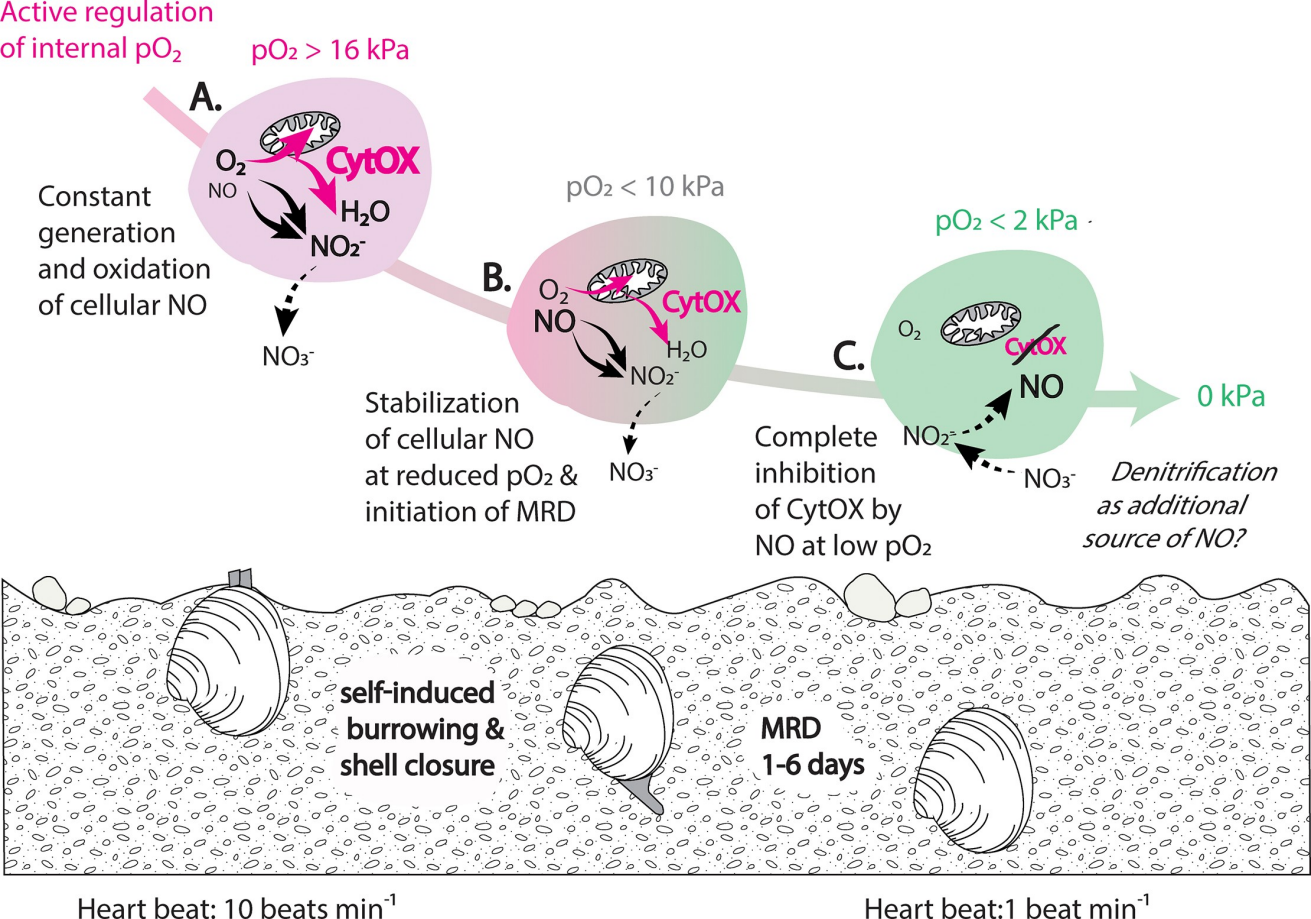
Potential role of nitric oxide (NO) in the mediation of metabolic rate depression (MRD) in *Arctica islandica* during self-induced burrowing and shell closure, when internal *p*O_2_ is actively down-regulated. A. At *p*O_2_ > 16kPa (pink background), standard metabolic rates are high (= high activity of cytochrome-c-oxidase, CytOX, fat pink arrows); cellular NO is constantly produced in the mitochondria and rapidly autoxidizes to nitrite (indicated by black arrows). B. During burrowing and shell closure, internal *p*O_2_ is down-regulated to < 10kPa and cellular NO starts to stabilize (pink/green background); NO induces a metabolic rate depression (MRD) by reversible inhibition of mitochondrial CytOx (small pink arrows). C. At *p*O_2_ < 2kPa (green background), NO reaches high stability and completely inhibits CytOx activity (CytOx crossed out); *Hypothesis*: *NO might form by acidic reduction of nitrite*, *originating from nitrate reduction by facultative anaerobic bacteria associated with tissues of A*. *islandica (dashed arrows and italic type)*, *which may lower the amount of ROS produced during anoxia/hypoxia-reoxygenation events at the cellular level*.

The results of the present study suggest that NO plays a role in metabolic down-regulation in *A*. *islandica* (Fig 7). At experimental *p*O_2_ ≤10 kPa, gill respiration of the ocean quahog was completely inhibited by concentrations between 0.7 and 1.9 μM NO, generated with the NO donor spermineNONOate in the incubation medium. The experimentally applied NO concentration was well below the calculated mean NO production for *A*. *islandica* hemolymph *in situ*. With an average production of 110 nM NO h^-1^ 100^−1^ hemocytes *in vitro* at 6 kPa, (which corresponds to the mean *p*O_2_ of *A*. *islandica in vivo* of ~ 5 kPa) [20], and based on a mean count of ~ 140,000 hemocyte cells ml^-1^ hemolymph [25], hemolymphatic NO production rates in *A*. *islandica* can be approximated to 154 μM NO h^- 1^.

In addition to the actual NO production, the stabilization of cellular NO by active down-regulation of internal *p*O_2_ is likely to be an important metabolic modulator in *Arctica* (Fig 7). Based on the calculated NO steady state ([NO]_steady state_, Table 3) in our tissue-free experiment, we showed that the removal of NO by oxidation increases by 70% between 2 and 16 kPa. Additionally, in our experiments with live gill tissue, NO was so rapidly oxidized at 16 kPa that even the 2–5 times higher spermineNONOate concentrations, which effected complete inhibition between 2 and 10 kPa, could not completely suppress the oxygen consumption of the gill pieces *in vitro*. In this context, it is interesting to note that the confocal image analysis of DAF-2T fluorescence revealed a largely stable NO signal for *Arctica* gill epithelia, independently of the experimental *p*O_2_ between 16kPa and 2kPa. Our results indicate that NO formation itself remains constant under normoxia and hypoxia in *A*. *islandica* gills. However, the active adjustment of mean internal *p*O_2_ to < 5 kPa in the animals *in vivo* [20, 24] appears to promote a stable [NO]_steady state_ in body fluids and tissues and the lowering of mitochondrial respiration by NO-induced CytOx inhibition (Fig 7B and 7C). The inhibitory effect of NO on multiple complexes of the electron transport chain is also well known from vertebrates. Poderoso and colleagues [36] found substantial inhibition of complex I (NADH-cytochrome c reductase), III (Ubiquinone-cytochrome b reductase) and IV (CytOx) in mitochondria of rats exposed to 0.1–0.3 μM NO, similar to the concentrations used in the present study. Thus, hemolymph NO stabilization at decreased internal *p*O_2_ could attenuate respiratory oxygen consumption and smooth out fluctuations of the internal *p*O_2_ in body fluids of *A*. *islandica* [20, 24]. Poderoso and colleagues hypothesized that reversible inhibition of CytOx activity and cellular O_2_ uptake by NO may decrease the steepness of the O_2_ gradient in the normoxia/anoxia transition zone of vertebrate organs [36]. In a cellular *p*O_2_ range between 1and 10 kPa, NO could delimit the intensity of mitochondrial respiration in *A*. *islandica* tissues against variable oxygen supply and achieve a more homogenous oxygenation of whole organs. Hence, NO may be instrumental in equilibrating mitochondrial electron flow against *p*O_2_ fluctuations and, in so doing, lower the amount of ROS produced during anoxia/hypoxia-reoxygenation events (e.g. surfacing, shell opening) at the cellular level.

### Chemical NO consumers in gill homogenates and excised tissues

Surprisingly, the amount of spermineNONOate necessary to achieve 50% inhibition of CytOx activity *in vitro* was 20 times higher at 2 kPa and 5 times higher at 6 kPa than the spermineNONOate concentration necessary to inhibit gill respiration at the respective *p*O_2_ (see Table 4 and Fig 6). Added to the tissue homogenate, NO is likely to react with freely accessible compounds such as metalloproteins (e.g. iron centers of proteins), thiols, disulfides, and diverse electron acceptors [37]. This chemical NO scavenging apparently increases the spermineNONOate concentration necessary to achieve inhibition of CytOx activity *in vitro*. Also, in intact gill pieces, NO can further react with superoxide anion radicals to peroxynitrite [8]. Superoxide anions were shown to form in considerable quantities in freshly excised gill tissue of *A*. *islandica* [20]. Peroxynitrite is a strong oxidant that lowers mitochondrial respiration by irreversible inhibition of complex I, complex II, CytOx, ATPase synthase and other enzymes [8, 38].

### Nitrite as a modulator of gill respiration

Nitrite has been suggested to inhibit aerobic respiration by interacting with CytOx in denitrifying bacteria [39]. Experimental exposure to 575 μM nitrite, a concentration well above the measurable NO_2_ levels in hemolymph (max. 37 μM), however, affected neither gill respiration rates nor *in vitro* CytOx activity of *A*. *islandica*. Our results suggest that the inhibitory effect might rather be due to NO that can also be generated from nitrite by non-enzymatic acidic reduction or by enzymatic reduction via xanthine oxidoreductase [12, 18, 19, 40], and not to a direct nitrite effect on animal cells and tissues.

### Possible sources of nitrate and nitrite in bivalve hemolymph under anoxia

In several studies, NO production of bivalve hemocytes is indirectly measured by quantifying its oxidation products nitrite (NO_2_^-^) and nitrate (NO_3_^-^) [3, 41, 42]. Our data suggest that the interpretation of nitrite and nitrate content in the context of NO formation in body fluids of molluscs requires caution, because nitrite and nitrate enrichment can originate from diverse sources in marine ecosystems, such as microbial activity, animal excretion, or simply the uptake from seawater or sediment pore water [43–45]. In fact, dissolved inorganic nitrogen (DIN = ammonium + nitrate + nitrite) of around 45 μM is common in coastal waters of the German Bight [46] and mean ∑NiNa determined in the incubation water (34 μM) and the hemolymph (37 μM) of *A*. *islandica* under normoxia match these values. Nitrate reduction by facultative anaerobic bacteria possibly associated with the body fluids of *A*. *islandica* may have caused the observed shift to nitrite after 3.5 days of anoxia, concurrent with the decrease of a corresponding amount of nitrate in the incubation water and the hemolymph (see also Table 2). This is convincing, as permanently open siphons in hypoxic and anoxia incubations allowed circulation and exchange of seawater in the mantle cavity of the clams. The microbiome of *A*. *islandica* has not yet been investigated, but literature studies show that microbiota of different genera colonize the hemolymph, intestine, shell and gills of bivalve mollusks [reviewed in 47]. Denitrifying bacteria forming part of the microbial biofilm of the shell surface, and the soft tissues of marine invertebrates [45, 47, 48, 49], and presumably also *A*. *islandica*, could reduce nitrate and nitrite under hypoxic and anoxic conditions. As an example, facultative anaerobic bacteria (e.g. *Vibrionaceae*) from seawater thrive in anaerobic conditions in the gut of the oyster, *Ostrea edulis*, fermenting carbohydrates and using nitrate as an alternative electron acceptor for their metabolism [45]. Furthermore, when the synthesis of nitrite reductase is depressed, or when nitrite reduction is inhibited by nitrate, some microorganisms involved in denitrification or nitrate assimilation can cause a rapid and massive accumulation of nitrite [50]. Such bacteria can be found in several vertebrates and invertebrates, i.e. in the gut of earthworms, where nitrite concentrations are 10-fold higher than in the environmental soils [51]. Further analysis is required to confirm the potential role of an associated microbiome in the production of nitrite, and potentially also NO (see next section), in the regulation of MRD in *A*. *islandica*.

Alternatively, *A*. *islandica* itself may possess nitrate reductase activity in its tissues, producing nitrite under anoxic conditions. Nitrate reductase activity has already been detected in cells of humans, mice and rats, and may represent a side reaction of xanthine oxidoreductase or of other enzymes [52]. Both prolonged hypoxia and acidosis increase the expression levels and the nitrate reducing capacities of xanthine oxidoreductase, suggestive of an increased nitrite formation under both conditions [52, 53].

### Nitrite and NO-signaling under anoxia–a hypothesis

While nitrite is usually considered to be an oxidative metabolite derived from NO, it can also be a storage pool for NO formation [54]. In mammals and ectotherms, NO can be generated from nitrite by non-enzymatic acidic reduction [18, 19, 55], or by enzymatic reduction via xanthine oxidoreductase [12, 52]. Both reactions are favored at low pH and low *p*O_2_ [18, 55], as well as at high c[NO_2_^-^] [19]. During prolonged anoxic incubation, or during shell valve closure in burrowing animals, intracellular acidosis may result from accumulation of anaerobic metabolites [55], likely leading to a highly reduced state that may also support the reduction of nitrite to NO (Fig 7C), comparable to NO generation during ischemia in mammals [18]. Thus, during prolonged anoxia in *A*. *islandica*, NO may function as a cellular protection mechanism, in addition to its assumed role in mediating MRD under hypoxia. When the bivalves surface after a period of burrowing, and tissues are re-oxygenated, NO could prevent high ROS production rates by CytOx inhibition and cellular O_2_ uptake. In contrast to several invertebrate species undergoing dormant states [56, 57], *A*. *islandica* neither exhibits a ROS-burst after hypoxia/reoxygenation nor up-regulates antioxidant capacities during prolonged MRD [20, 26].

## Conclusions

In conclusion, NO has several functions for respiratory regulation in this hypoxia- and anoxia-tolerant clam, including mediation of metabolic down-regulation at internal *p*O_2_ levels < 10 kPa during self-induced burrowing and shell closure. Hemocyte cells, as well as mitochondria and tissue filaments of the gill of *A*. *islandica*, produced measurable amounts of NO, with no clear indication of NOS-like (and L-NAME inhibitable) activity, which might be typical for hypoxia tolerant deep burrowing clams. Externally applied NO in the μM range, similar to NO concentrations in the hemolymph of *A*. *islandica*, significantly inhibited gill respiration and CytOx activity. A controlled metabolic shutdown, by reducing electron transport at complex I and oxygen binding at complex IV of the respiratory chain, may also prevent an extensive production of ROS during surfacing and reoxygenation after prolonged hypoxia and anoxia. Further physiological and genetic studies are needed to identify possible enzymatic and/or non-enzymatic NO forming processes in hemocytes and tissues of *A*. *islandica*. High nitrite concentrations in the anoxic hemolymph point towards nitrate reduction by facultative anaerobic bacteria, possibly associated with body fluids, organs and/or tissues of *A*. *islandica*. This, however, needs verification, and future studies should focus more on the role of associated microbial biofilms in the mediation of physiological functions in marine bivalves.
